# The Mechanism and Potential Therapeutic Effects of Cyclosporin, Cyclophilin, Probiotics and Syndecan-1 in an Animal Model of Inflammatory Bowel Disease

**DOI:** 10.3390/pharmaceutics16010130

**Published:** 2024-01-19

**Authors:** Laura Dosh, Francesca Rappa, Abdo Jurjus, Gaelle Karam, Roaa Lezeik, Jad El Masri, Fabio Bucchieri, Angelo Leone, Rosalyn Jurjus

**Affiliations:** 1 Department of Anatomy, Cell Biology and Physiological Sciences, Faculty of Medicine, American University of Beirut, Beirut 1107-2020, Lebanon; 2 Department of Biomedicine, Neuroscience and Advanced Diagnostic, University of Palermo, 90127 Palermo, Italy

**Keywords:** cyclosporine A, cyclophilin A, probiotics, IBD, inflammation, syndecan-1

## Abstract

**Background:** Inflammatory bowel diseases (IBDs) have several treatment modalities including immunoregulators, like cyclosporine A, an immunosuppressant that interacts with cytoplasmic cyclophilin A, and probiotics. **Aims:** This study explored and compared the possible role of syndecan-1 in the IBD pathogenic process as well as the effectiveness of cyclophilin A, cyclosporine A, and their combination in the management of IBDs in the presence of probiotics. **Methodology:** IBD was induced in a total of 112 mice equally divided between **syndecan-1** knock-out (KO) and Balb/c wild-type mice, using 2% dextran sulfate sodium (DSS) followed by intraperitoneal treatment with cyclosporine A, cyclophilin A, or a combination of both. In addition, a daily dose of probiotics was given in their drinking water. The animals were monitored for clinical signs and symptoms and checked for gross pathologies in the abdomen after 3 weeks. Descending and sigmoid colon biopsies were collected and fixed for routine microscopy or frozen for protein extraction and molecular testing for IL-6, CD3, CD147, and beta 1 integrins as well as pAkt expression. **Results:** The data showed that the induction of IBD in the syndecan-1 KO mice was more severe at the clinical, histological, and molecular levels than in the wild type. The combined CypA-CyA treatment showed no added inhibitory effect compared to single-drug treatment in both strains. Probiotics added to the combination was more effective in the wild type and, when used alone, its inhibition of IL-6 was the highest. As for the CD147 marker, there were more suppressions across the various groups in the KO mice except for the probiotics-alone group. Concerning CD3, it was significantly increased by the CypA-CyA complex, which led to more inflammation in the KO mice. Probiotics had little effect with the combination. In relation to beta 1 integrins, the CypA-CyA combination made no significant difference from CyA alone, and adding probiotics to the combination resulted in higher beta 1 integrin expression in the KO mice. As for pAkt, it was very well expressed and upregulated in both strains treated with DSS, but the effect was much larger in the KO mice. In brief, the CypA-CyA complex showed a decrease in the expression of pAkt, but there was no added effect of both drugs. Probiotics along with the complex had a similar reduction effects in both strains, with a greater effect in the wild-type mice, while probiotics alone led to a similar reduction in pAkt expressions in both strains. **Conclusions:** The differential effects of CyA, CypA, probiotics, and their combinations on the various inflammatory markers, as well as the histological alterations and clinical signs and symptoms, speak in favor of a clear role of syndecan-1 in reducing inflammation. However, probiotics need to be considered after more explorations into the mechanisms involved in the presence of CypA and CyA especially since pAkt is less active in their presence.

## 1. Introduction

Inflammatory bowel diseases (IBDs) constitute a continuum of refractory autoimmune inflammatory disorders ranging from ulcerative colitis (UC) to Crohn’s disease (CD) [1]. Inflammation is the basic underlying mechanism of these disorders, yet the exact etiology of IBDs has not been fully elucidated [2]. Studies have documented that IBDs are the result of a plethora of interactions of genetic and non-genetic parameters involving the microbiome, the environment, and the immune system, which plays a crucial role in initiating an excessive inflammatory response in genetically susceptible individuals [3,4]. Consequently, in this process, a panoply of pro-inflammatory and anti-inflammatory secretions interact through various signaling pathways [5]. 

Moreover, cell adhesion molecules (CAMs) have been implicated in the pathogenesis of IBDs, as a reduction in epithelial matrix adhesion is likely to cause decreased healing [6]. For example, lower levels of syndecan-1 were found in the colon epithelium of IBD patients [7]. In addition, the microbiome, whether dysbiotic or eubiotic, is an essential player in such a pathogenic process, as demonstrated in multiple human and animal studies [8].

Accordingly, the treatment targets and modalities of IBDs have profoundly changed over recent years following discoveries of the multiple pathogenic pathways involved; currently, they are stressing healing rather than symptom control [9]. On this basis, various management protocols for IBD included immunomodulators like cyclosporine and anti-cytokine antibodies like anti-TNF-α and anti-IL23 [9]. In addition, the approach of using probiotics has been adopted in multiple instances with encouraging results both in animal and human studies but never in the presence of cyclosporine [10]. Cyclosporine A (CyA), an immunosuppressant that interacts with cytoplasmic cyclophilin A (CypA) [11,12], was introduced in the mid-1980s. In addition, probiotics have been used in the last two decades to correct dysbiosis [13].

IL-6 has a significant role in the etiology of IBDs through a pro-inflammatory effect [14,15]. Similarly, CD3 and CD147 are upregulated in IBDs, playing essential roles in modulating inflammation [16,17]. The activation of the PI3K/Akt/mTOR pathway in lymphocytes also leads to their activation and, hence, leads to IBD [18]. Furthermore, many of the key cell–cell and cell–matrix interactions are regulated by beta integrins, whose deficiency may significantly enhance both the pathogenesis and development of IBDs [19]. 

The data from the literature report that each of the adopted management protocols has not been without limitations and drawbacks. For instance, CyA exhibited several complications in terms of toxicity to various body organs [20]. Accordingly, it was believed that the administration of the CypA compound that interacts with CyA would enhance its immunosuppressant effect and probably decrease its side effects [21]. 

Knowing that the absence of syndecan-1 affects the gut microbiota and disrupts the homeostasis of microbes internally, increasing the likelihood of IBD development, and supported by data regarding polymorphisms in IBD susceptibility genes, this study explored and compared the IBD pathogenic process in syndecan-1 KO mice compared to wild-type Balb/c mice [22,23,24]. Such an approach to IBDs will possibly pave the way to understanding the many mechanistic pathways involved in maintaining intestinal homeostasis. It will also show the potential difference in DSS-IBD induction between various mice strains and the possible role of syndecan-1 in such a process. Furthermore, this study assessed the effectiveness of CyA in the management of IBDs and the potential role of CypA in enhancing the healing process of an inflamed gut, particularly in the presence of probiotics. In brief, it highlights the potential role of probiotics in IBDs in syndecan-1 KO mice compared to wild type.

## 2. Materials and Methods

### 2.1. Animals

A total of 112, 6–8-week-old male mice were divided into 2 main groups: Wild-type Balb/c mice and KO Syndecan-1 null mice (56 mice of each strain). All the animals were housed in the Animal Care Facility of the American University of Beirut (AUB). All animal experiments and procedures strictly followed the guidelines of the Institutional Animal Care and Use Committee (IACUC) at the American University of Beirut for the care and use of laboratory animals. 

### 2.2. Experimental Design

A total of 16 control mice—8 from each strain—were only provided with normal drinking water and intraperitoneal (IP) saline injections. IBD was induced in 48 animals from each strain (96 mice) by 2% DSS in drinking water. Each DSS cycle consisted of 7 days of DSS followed by 2 weeks of normal drinking water. At week 3, the animals were sacrificed. 

The 96 mice with DSS induced IBD were divided according to the different treatment modalities into a total of 12 subgroups (8 animals per group). Treatments were administered as illustrated in Table 1. 

### 2.3. Induction of IBD and Treatments

Optimized concentration of the pro-inflammatory agent dextran sodium sulfate (DSS; Sigma-Aldrich, St. Louis, MO, USA, 42867-100G) 2% was prepared in autoclaved water and administered to animals in their drinking water. Each DSS cycle consisted of 7 days of DSS followed by 2 weeks normal drinking water and treatments. 

Cyclophilin A (human recombinant expressed in *E-coli*) from Sigma-Aldrich (C3805-1MG) was injected intraperitoneally (IP) at a dose of 25 µg/kg/day for two weeks starting day 7 of DSS administration. Similarly, cyclosporine A (Novartis, Basel, Switzerland, SPE31) was administered by IP injections at a concentration of 200 µg every other day for 2 weeks starting day 7 of DSS treatment. In addition, the probiotics (P) used was a mixture of 7 strains of lactic acid-producing bacteria: *Lactobacillus rhamnosus*, *Saccharomyces boulardii*, *Bifidobacterium breve*, *Bifidobacterium lactis*, *Lactobacillus acidophilus*, *Lactobacillus plantarum* and *Lactobacillus reuteri.* One capsule of (P) was dissolved in 1.75 L of autoclaved tap water to reach a daily dose of 10^8^ CFU per animal and given for 2 weeks starting day 7 of DSS treatment. 

### 2.4. Clinical Course Assessment

During the experimental period, the animals were monitored daily for clinical symptoms and signs including body weight, stool aspect, and rectal bleeding. Scores were recorded and calculated throughout the experiment based on the parameters: gross bleeding (0 = absence; 2 = blood stained; 4 = clear presence of blood), stool consistency and watery diarrhea (0 = normal, 2 = loose, 4 = diarrhea), weight loss (0 = normal; 1 = 1–5%; 2 = 5–10%; 3 = 10–20%; 4 more than 20%), and a previously validated clinical disease activity index, (DAI) which assesses weight loss, diarrhea, fur quality, anal bleeding and posture with a range of 0 to 4 calculated [25]. 

### 2.5. Measurement of Fecal Occult Blood

Collection of feces was completed by placing a single mouse in an empty cage without bedding material for a few minutes; feces were collected, and occult blood was measured using HemoCue America Beckman Coulter™ Hemoccult™ Fecal Occult Blood Slide Test System (Beckman Coulter, Brea, CA, USA), as per the manufacturer’s instructions [26]. 

### 2.6. Dissection, Colon Length Measurement and Biopsy Removal

After 3 weeks, at the experiment endpoint, animals were sacrificed by isofurane overdose and cervical dislocation; then, they were dissected in order to remove their colon. We measured and recorded the length of each isolated colon from the ileocecal valve to the rectum using a ruler; then, it was quickly flushed on ice with phosphate-buffered saline (PBS) to clean it. A portion of this clean colon, the sigmoid, was fixed in 10% buffered formalin for routine histological processing. The other portion of the sigmoid and the descending colon were frozen and kept in liquid nitrogen for further molecular studies. 

### 2.7. Histology

Biopsies were embedded in paraffin and seven slides of each biopsy, each having 2 sections, were stained either with hematoxylin and eosin (H&E) or periodic acid–Schiff (PAS). The slides were finally scanned using a light microscope. The histological score of each tissue section of each animal within each group was assessed and calculated based on a histological scoring system shown in Table 2 [27]. The different sections were photographed using an Olympus CX-41 microscope (Olympus, Tokyo, Japan). 

### 2.8. Western Blot

Western blotting analysis was performed using 100 mg of the frozen colon tissue according to standard protocols. Protein concentration was determined by the Lowry method using the DC™ Protein Assay Kit (#5000111, BIO-RAD, Hercules, CA, USA). Antibodies against IL-6 (anti-mouse SC-57315), CD147 (anti-mouse SC-46700), Actin (anti-mouse SC-47778), CD3 (anti-mouse 20047), and pAKT (anti-mouse SC57315) were detected using a secondary antibody (horseradish peroxidase-conjugated anti-mouse; Abcam 97,046 at a dilution of 3:40,000 (Abcam, Boston, MA, USA)). The immunoprecipitated protein bands were detected with ChemiDoc MP Imaging System-Biorad (BioRad, Hercules, CA, USA).

### 2.9. Immunohistochemistry

Immunohistochemistry was performed on 5-micron thick paraffin embedded sections. Slides were stained with a primary antibody (anti-integrin ß1-JB 1B sc-59,829 mouse IgG from Santa Cruz Biotechnology, Dallas, TX, USA). Quantification was performed by obtaining the measurement of mean fluorescence intensity (MFI) of integrin ß1 in a region of interest (ROI) and calculating the integrated density using Zen 2.3 Software (Carl Zeiss Microscopy GmbH, Oberkochen, Germany) [28].

### 2.10. Statistical Analysis

Statistical analysis was performed using GraphPad Prism 8.0.1. Data were expressed as a mean ± standard deviation. Significant differences were evaluated using the one way ANOVA by the Tukey–Kramer multiple comparisons test. A value of *p* < 0.05 was considered significant. 

## 3. Results

### 3.1. Macroscopic Assessment

#### 3.1.1. Clinical Symptoms and Signs

In wild-type group 2, when DSS was used alone, six out of eight animals showed severe diarrhea and five suffered from hematochezia; in comparison, eight out of eight of the syndecan-1 KO mice showed severe diarrhea, and six mice suffered from hematochezia (Table 3). 

When cyclophilin A (Cyp A) was added alone, group 3, hematochezia and diarrhea were both present in five mice out of eight in the wild-type mice, while seven of the syndecan-1 KO mice showed diarrhea and five suffered from hematochezia (Table 3). 

Cyclosporine (CyA) alone, group 4, reduced the incidence of hematochezia in the wild type from five to three mice only and diarrhea from six to four mice. However, for the syndecan1 KO mice group 4, diarrhea was present in three mice and hematochezia was present in four mice compared to six mice in group 2 when DSS was used alone (Table 3). 

On the other hand, treatment with the CypA-CyA combination, group 5, showed hematochezia in three mice instead of five in the DSS-only group for the wild type, but no effect was noted on diarrhea, which remained in six mice. However, in the KO mice with the combination of CypA-CyA, hematochezia was still detected in four mice and diarrhea was detected in three mice (Table 3). 

When adding probiotics to the combination, group 6, two mice showed hematochezia in the wild type compared to five mice in group 2, which were given DSS only, and four mice suffered from diarrhea. On the other hand, three KO mice suffered from hematochezia and three suffered from diarrhea when given the CypA-CyA combination plus probiotics (Table 3). 

Treatment with probiotics alone, in mice with DSS induced IBD, stopped the bleeding in all mice from both strains and reduced diarrhea to two animals only in the wild type and two as well in sydecan-1 KO mice (Table 3). 

As for the DAI, the highest disease activity indices were obtained in animals belonging to the DSS-only group in both strains. It is important to note that treatment with probiotics alone improved the clinical profile and decreased the DAI to almost 0 in the wild-type mice. On the other hand, in syndecan-1 KO mice, DSS alone and DSS plus the combination of cyclosporine and cyclophilin showed the highest DAI, and mice were shown to present more signs of discomfort and distress. 

#### 3.1.2. Shortening of the Colon

**Figure 1:**
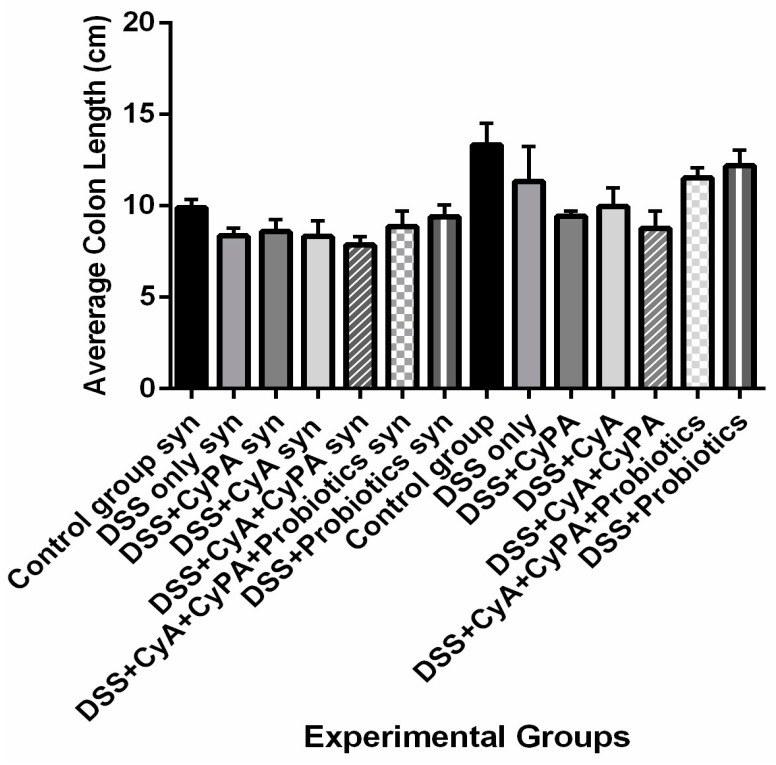
Colon length variation among the various groups at the day of sacrifice.

DSS treatment shortened the colon by a ratio of 8/10 = 20% in Sydecan1 KO mice and 11.2/13.4 = 16.5% in the wild type compared to the controls (Figure 1). CyA and CypA did not prevent this shortening; they actually enhanced it slightly and more so when present in combination without probiotics (in particular in the wild type). However, the shortenings were only slightly significant when compared to the control non-treated group, in particular, in the wild type; they might need more chronic cases to show more difference.

In brief, probiotics used alone or in combination with the CypA-CyA complex prevented to a great extent such shortenings and more so in the wild type, which is where the shortening was more obvious. 

### 3.2. Microscopic Assessment

#### 3.2.1. Assessment of Colon Histology by H&E Stain

##### Wild-Type Group

**Figure 2:**
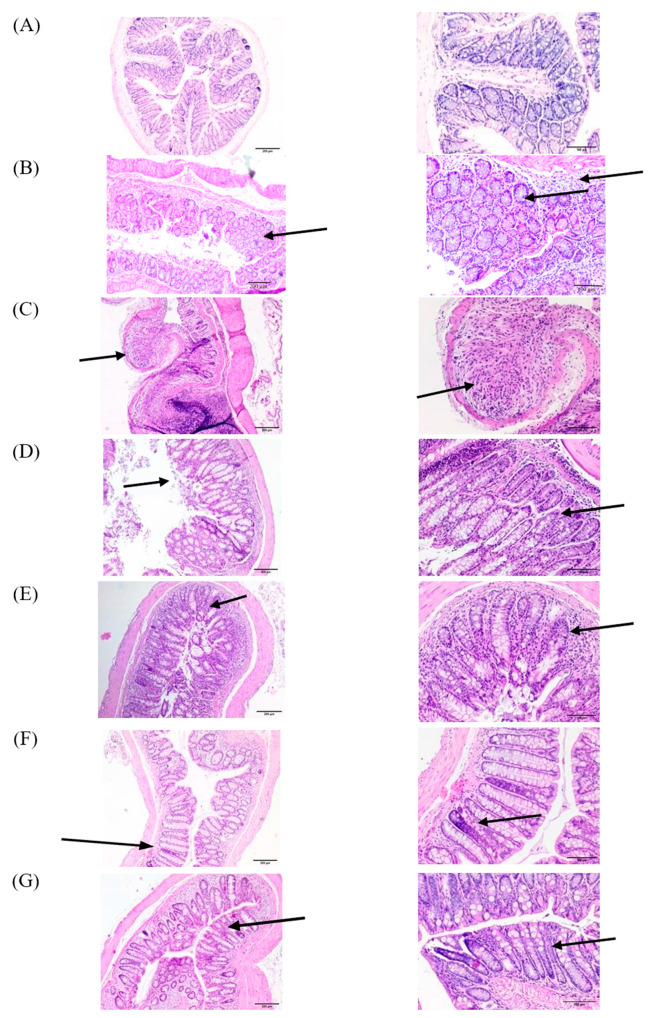
Wild-type mice: effect of cyclophilin A, cyclosporine A and probiotics on colon histology (H&E). Slides were stained with H&E and photographed at 40× magnification, scale bar 200 micron ((**left**) column) and 100×, scale bar 100 micron ((**right**) column). Arrows indicate reaction sites. (**A**) Negative control group (no DSS); (**B**) DSS-treated group; (**C**) DSS + CypA-treated group; (**D**) DSS + CyA-treated group; (**E**) DSS + (CypA-CyA) complex treated; (**F**) DSS + (CypA-CyA) complex + probiotics-treated group; (**G**) DSS + probiotics group.

Concerning the wild type, as shown in Figure 2A, the histological architecture of the colon in the healthy control group 1 showed no sign of disrupted morphology and a normal histological appearance (Table 4, group 1).

The DSS-treated group 2 (Figure 2B) exhibited an extensive inflammatory cell infiltration that invaded the mucosa and submucosa and reached the muscular layer. A disruption in integrity of the crypts and a loss of epithelial lining were also observed (Figure 2B, group 2). Similarly, the group treated with CypA displayed high inflammatory cell infiltration, disorganized crypts, and loss of the majority of the epithelial lining (Figure 2C). However, colonic inflammation subsided significantly in the groups treated with either CyA (Figure 2D), probiotics (P) (Figure 2F), or their combination (CyA + CypA; CyA + CypA + P) (Figure 2E and Figure 2G, respectively). The mucosal architecture, the crypt integrity and the epithelial lining were restored to a great extent. Inflammatory cells activity was significantly reduced compared to the DSS-treated group 2.

##### Syndecan-1 KO Group

**Figure 3:**
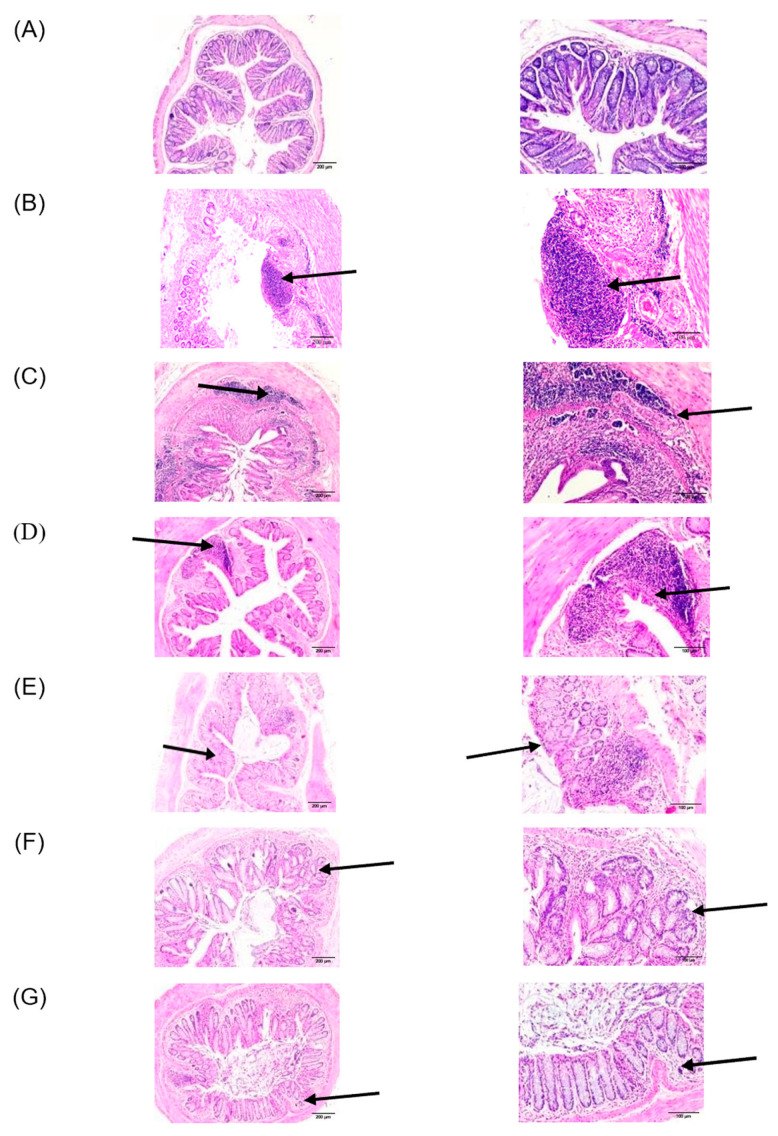
Syndecan-1 null mice: effect of cyclophilin A, cyclosporine and probiotics on colon histology (H&E). Slides were stained with H&E and photographed at 40× magnification, scale bar 200 micron ((**left**) column) and 100×, scale bar 100 micron ((**right**) column). Arrows indicate reaction sites. (**A**) Negative control group (no DSS) showing normal colon morphology with normal crypt architecture; (**B**) DSS-treated group showing a marked localized inflammation invading mucosa and submucosa and characterized by edema, massive increase in leukocyte infiltration. However, semi-preserved crypts are present throughout the remaining areas with low infiltration. (**C**) DSS + CypA-treated group showing a mild inflammation with an improvement in the colonic histology, preservation of overall architecture, and low leukocyte infiltration. (**D**) DSS + CyA-treated group showing a mild inflammation with only a one-third crypt damage and very active Peyer’s patches. (**E**) DSS + (CypA-CyA) complex-treated group showing a detrimental destruction of the complete colonic morphology and crypt architecture with an intensive leukocyte infiltration. (**F**) DSS + (CypA-CyA) complex + probiotics-treated group showing a moderate inflammation extending from submucosa to mucosa with a one-third damage of the crypts. A heavy leukocyte infiltration is restricted to the space between the muscular layer and submucosa in addition to mucosa. (**G**) DSS + probiotics group showing a marked reduction in inflammation where only one-third of the crypts is distorted. However, a moderate leukocyte infiltration is concentrated at the lymphocytes aggregates, while the remaining areas reflect a normal morphology with a low infiltration.

In general, a more severe inflammation was observed in the DSS group 2S (Figure 3B); such inflammation extended from mucosa to submucosa layers with the presence of a marked edema between the muscular layer and submucosa. A massive increase in immune cell infiltration and a complete loss of epithelial architecture were both observed. However, some portions of the section were characterized by semipreserved crypts with a low leukocyte Infiltration (Figure 3B). 

On the other hand, mice treated with DSS + CypA (group 3S) and DSS + CyA (group 4) exhibited a significant improvement in colonic histology, showing a mild inflammation compared to the group treated with DSS only (group 2S; Figure 3C,D). Only one-third of the damage to the crypts is present with a very low level of edema between the muscular layer and submucosa. In brief, the overall architecture was almost preserved with a low level of leukocyte infiltration localized in the mucosa and submucosa layers (Figure 3D). 

The treatment with (CypA-CyA) complex group 5S along with DSS caused a detrimental effect on the tissue architecture where two–thirds of the crypts are lost in addition to the intensive leukocyte infiltration spreading throughout the mucosa and submucosa. The thickening of the mucosa and submucosa layers was significant as well as the notable presence of areas of complete epithelial denudation (Figure 3E). 

On the other hand, a slight reduction in the severity of inflammation was clearly observed after the addition of probiotics to the CypA-CyA complex along with DSS (Figure 3F). However, the group treated with DSS + probiotics showed only a marked reduction in inflammation in the absence of the CypA-CyA complex (Figure 3G). 

### 3.3. Assessment of Mucus Secreting Cells, Goblet Cell, by Periodic Acid Schiff Stain (PAS)

#### 3.3.1. Wild-Type Group

The group that received DSS showed a very significant (almost 100%) loss of goblet cells and a change in their architecture. The group treated with CypA displayed a loss of more than 60% of goblet cells with the presence of inflammation. However, goblet cells have been partially restored in the groups treated with CyA where around 70% of goblet cells were present and there was less inflammation.

Similarly, the group treated with DSS and the CypA-CyA complex showed hyperactivity and inflammation with almost 50% loss of goblet cells. When probiotics were added to the complex, mild progress was noticed to goblet cells, and inflammation was still persistent. On the other hand, when a probiotic was given alone with DSS, 80% of goblet cells were retained, and the architecture almost returned back to normal. 

#### 3.3.2. Syndecan-1 KO Group

A loss of almost 80–90% of goblet cells is detected in the DSS-treated group. In contrast, in the group treated with DSS + CypA, more than 75% of goblet cells were present throughout the tissue, and about 25% only have been lost. Similarly, the same effect has been revealed in the group treated with DSS + CyA, where only 25% of goblet cells were lost. On the other hand, the group treated with DSS plus the CypA-CyA complex presented a complete loss of goblet cells, while the group treated with DSS plus the CypA-CyA complex and probiotics retained about 25% of goblet cells. In contrast, the treatment of DSS and probiotics only in the absence of the complex has evidently improved the colonic histopathological state, retaining about 75% of goblet cells. 

**Figure 4:**
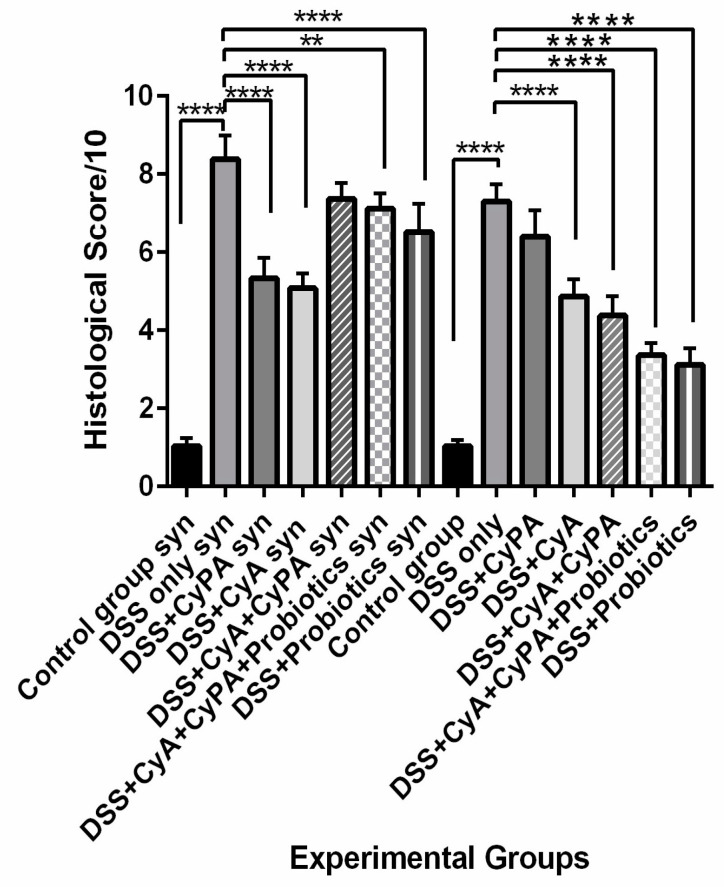
Histological alterations score. Effect of cyclophilin A, cyclosporine A and probiotics on colon histology in both strains. In syndecan-1 null mice, the negative control group (no DSS) shows a score of (1.04/10), the DSS group shows a score of (8.38/10). The DSS + CyPA and DSS + CyA groups have the same histological score (around 5/10). The DSS + CyPA-CyA complex group shows a high score of 7.36/10. The DSS + CyPA-CyA complex + probiotics group shows a score of 7.12/10 and the DSS + probiotics group shows a score of 6.52/10. On the other hand, in bulb/c mice, the negative control group (no DSS) shows a score of 1.03/10, the DSS group shows a score of 7.3/10. The DSS + CyPA group shows a score of 6.4/10 and the DSS + CyA group shows a score of 4.8/10. The DSS + CyPA-CyA complex group shows a score of 4.36/10. The DSS + CyPA-CyA complex + probiotics group shows a score of 3.36/10 and the DSS + probiotics group shows a score of 3.12/10. Statistical significance is determined by one-way ANOVA. *p* < 0.01 is indicated by (**), and *p* < 0.0001 is indicated by (****). Data are expressed as mean ±SEM (*n* = 8).

Histological scoring confirmed the above-mentioned observational findings, indicating that inflammation was most significantly alleviated in groups treated with the combinations (CyA + CypA + P or CyA + CypA), probiotics and cyclosporine, compared to those in the DSS and cyclophilin-treated groups (Figure 4).

### 3.4. Modulation in Inflammatory Markers

**Figure 5:**
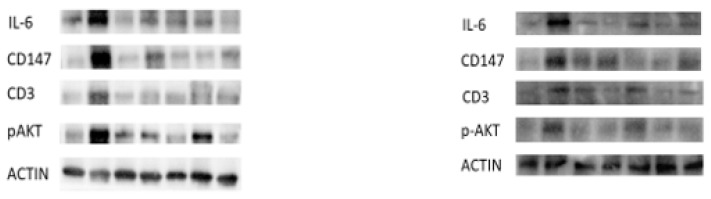
Representative Western blot bands for the expressions of IL-6, CD147, CD3, P-AKT and actin in Balb/c mice ((**left**) column) and in syndecan-1 mice ((**right**) column).

The lack of syndecan-1 led to an exacerbation in the inflammation induced by DSS. The tested molecular markers showed a significant differential in their respective expressions between wild type and the KO strains with respect to IL-6, 147, and CD3 for beta integrin and pAKT (Figure 5).

CyA, CypA and probiotics modulated significantly the expression of some inflammatory molecular markers: IL-6, CD147, CD3, beta 1 integrins and pAkt (Figure 5). There was a marked inhibition of the IL-6 by CyA and CypA when used separately with KO compared to wild type. However, this effect was reduced when the combination of CyA-CypA was used (Figure 5).

#### 3.4.1. IL6

**Figure 6:**
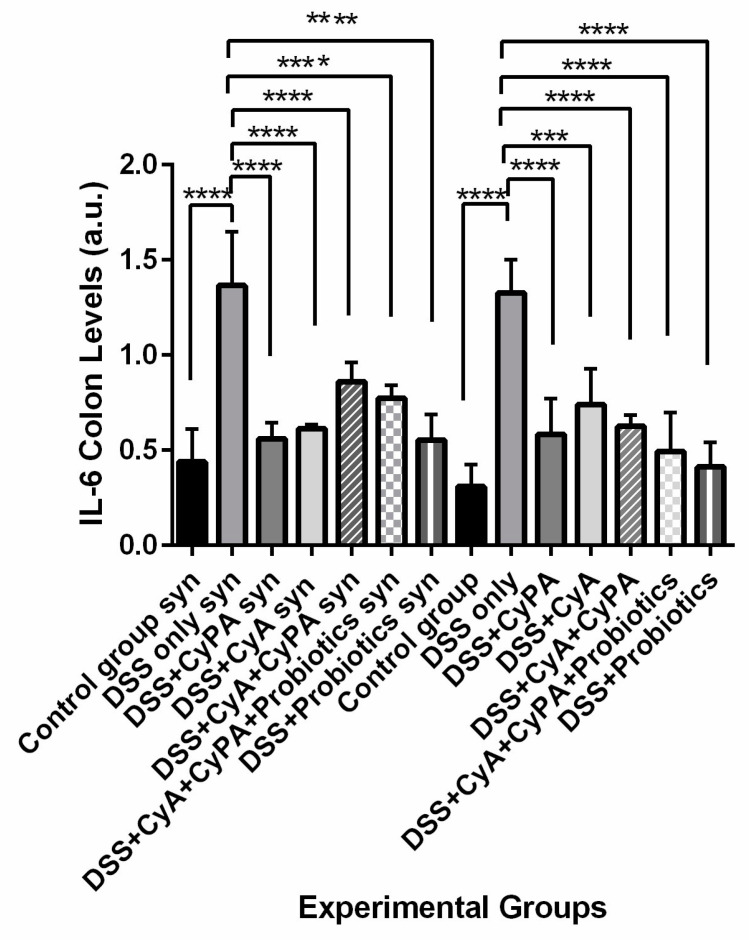
Variation of IL-6 levels evaluated by Western blot technique from colon extraction of the different experimental groups. *p* < 0.001 is indicated by (***) and *p* < 0.0001 is indicated by (****).

CypA and CyA alone inhibited the expression of IL-6 compared to the DSS alone to significant extents in syndecan-1 KO and wild type. When combined together, the CypA-CyA complex was less effective in decreasing the level of IL-6 expression in the syndecan-1 KO. In the wild type, IL-6 expression was less affected by the complex. Furthermore, the probiotics had less effect in reducing IL-6 expression in syndecan-1 KO compared to wild type both in combination with the CypA-CyA or separately (Figure 6).

#### 3.4.2. CD 147

**Figure 7:**
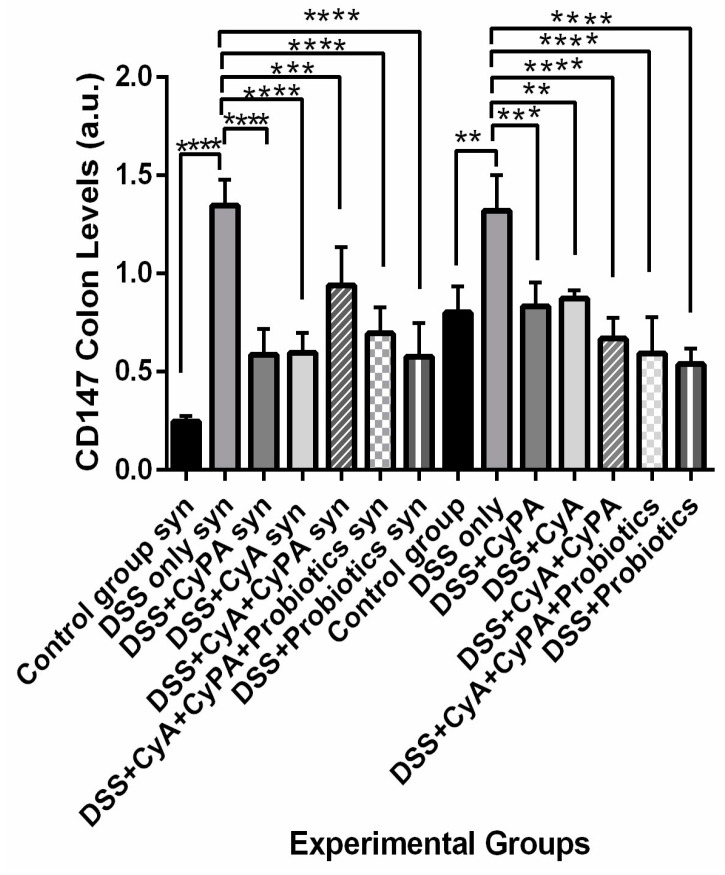
Variation of CD147 levels, evaluated by Western blot technique, from colon extraction of the different experimental groups. *p* < 0.01 is indicated by (**), *p* < 0.001 is indicated by (***) and *p* < 0.0001 is indicated by (****).

The CypA and CyA decrease the expression of CD147 significantly compared to the DSS group more in the syndecan-1 KO compared to the wild type. However, when the complex CypA-CyA was the treatment, the expression of CD147 increased more in syndecan-1 KO compared to the wild type and to each drug separately. The use of probiotics alone maintained a lower expression of CD147 similar in both strains with a slightly lower effect in the KO mice. However, adding probiotics to the CypA-CyA combination improved the inhibitory effect on CD147 expression in both strains compared to the CypA-CyA combination without probiotics with ratios of 51 in KO mice and 45 in wild type, i.e., more suppression in the wild type (Figure 7). 

#### 3.4.3. CD3

**Figure 8:**
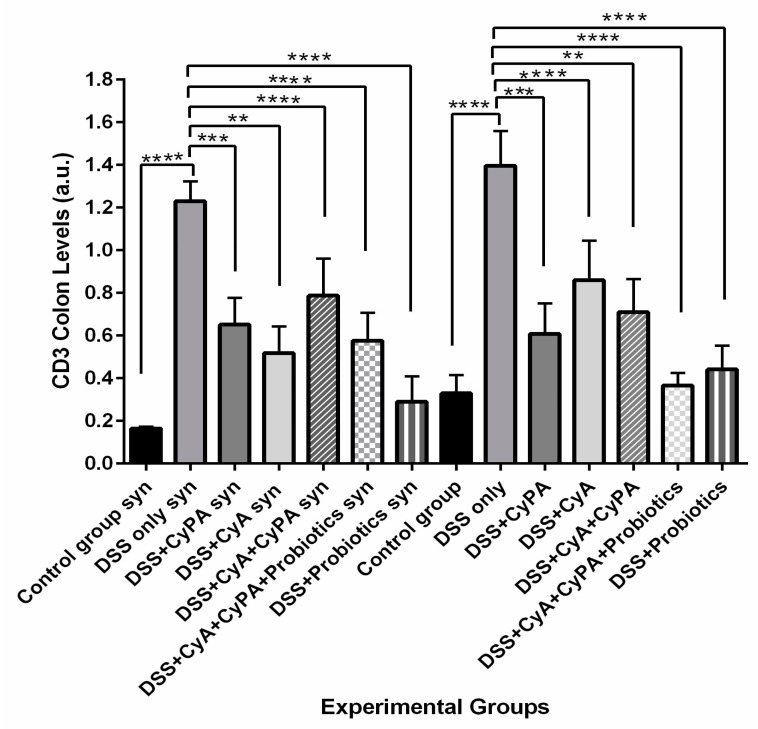
Variation of CD3 levels, evaluated by Western blot technique, from colon extraction of the different experimental groups. *p* < 0.01 is indicated by (**), *p* < 0.001 is indicated by (***) and *p* < 0.0001 is indicated by (****).

Treatment with CypA and CyA decreased the CD3 expression compared to the DSS group with a more significant decrease in CD3 expression in the KO mice. The use of the CypA-CyA combination did not have any added value for the suppression of CD3 expression, where it increased the expression in the KO mice compared to either CypA or CyA alone. Adding probiotics to the CypA-CyA combination lowered the CD3 expression compared to the combination without probiotics, where the effect of probiotics in the combination differed between the two strains when added to the combination; it was more effective in suppressing CD3 in the wild type. On the other hand, both strains responded relatively well to the probiotics treatment alone and decreased the expression of CD3 to significant levels compared to the DSS-alone group (Figure 8). 

#### 3.4.4. β1 Integrin

**Figure 9:**
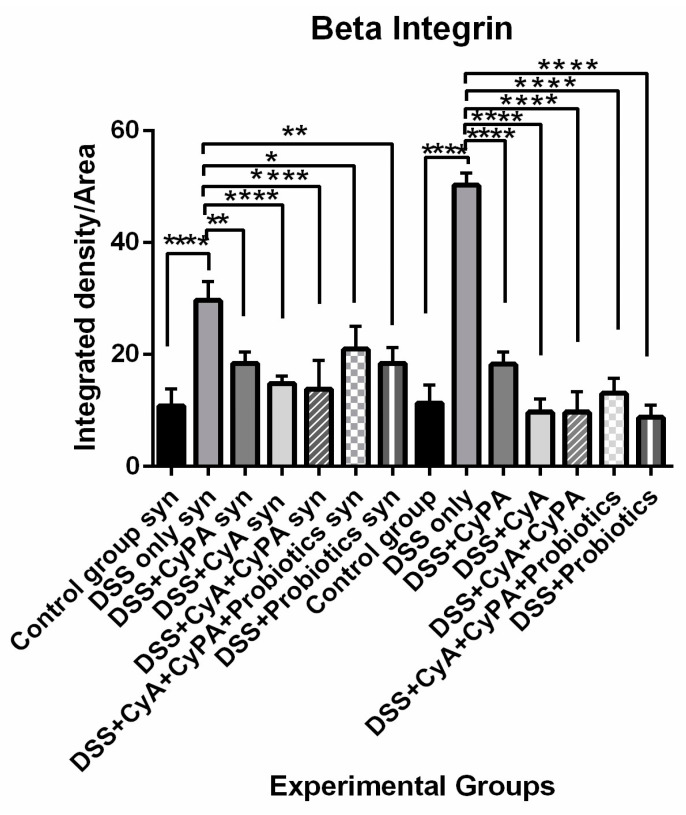
Variation of ß 1 integrin levels, assessed by immunofluorescence technique, of the different experimental groups. *p*-value < 0.05 is considered significant and is indicated by (*), *p* < 0.01 is indicated by (**), and *p* < 0.0001 is indicated by (****).

Treatment with CypA and CyA significantly decreased the beta 1 integrin expression in both strains but more so in the wild type, which showed almost 25% less expression in the KO mice. The suppression of expression was very much more significant in the wild type compared to the KO mice. However, treatment with the combination of CypA-CyA did not add to the reduction in expression produced by each alone in both strains. On the other hand, adding probiotics to the CypA-CyA combination led to a high expression of beta 1 integrin in both strains, rather than reduction, which was way beyond the combination of CypA-CyA or any drug alone. On the other hand, probiotics alone did reduce significantly the expression of beta 1 integrin in both strains but less so in the KO mice (Figure 9). 

#### 3.4.5. pAKT

**Figure 10:**
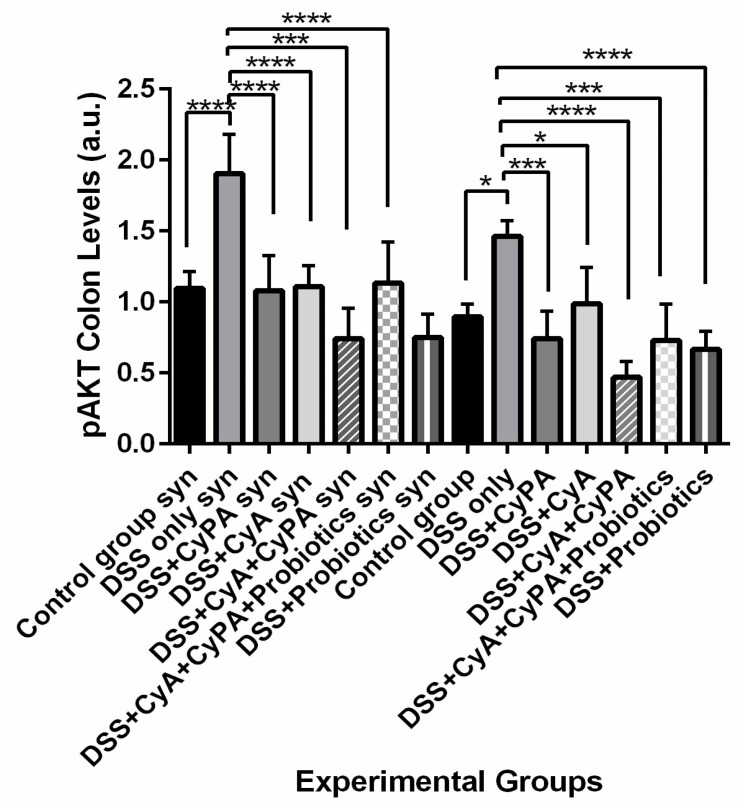
Variation of pAKT levels, assessed by Western blot technique, from colon extraction of the different experimental groups. *p*-value < 0.05 is considered significant and is indicated by (*), *p* < 0.001 is indicated by (***) and *p* < 0.0001 is indicated by (****).

In both mice groups, the treatment with CypA or CyA reduced the expression of pAkt to the level of control. This decrease in pAkt expression was further reduced when the combination was used. However, adding probiotics to the combination brought back a similar effect to either compound alone. On the other hand, probiotics alone behaved very much like the combination. In wild-type case, the combination as well as the probiotics decreased pAkt expression significantly in both strains to similar extents (Figure 10).

Table 5 and Table 6 summarize the clinical, histological and immunological (molecular) findings with CypA or CyA alone, and with their combination, in wild-type and in KO mice.

**Figure 11:**
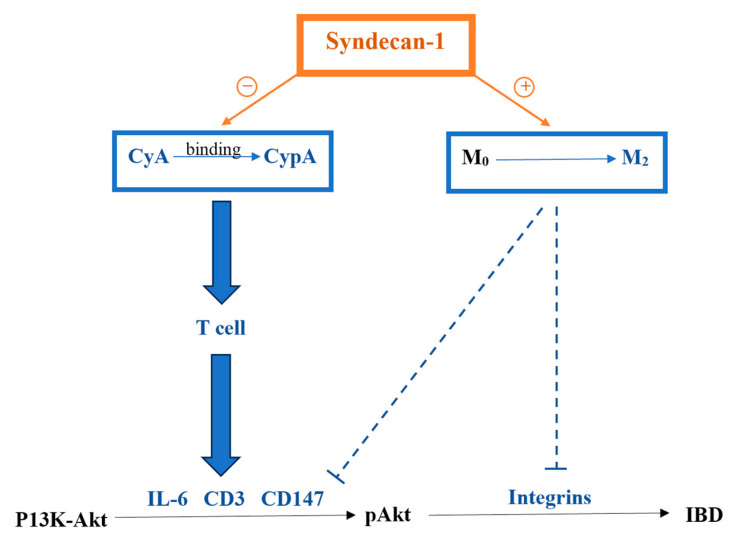
Schematic diagram clarifying the role of syndecan-1 in IBD pathogenesis. Syndecan-1 prevents the binding of CyA to CypA, which in turn enhances the effect of IL-6, CD3 and CD147 by acting on T cells, and it enhances the transformation of M0 to M2, enhancing its inhibitory effects on IL-6, CD3, CD147, and integrins. Syndecan-1 plays an inhibitory role in the pathogenesis of IBD through these 2 pathways.

Figure 11 represents a schematic diagram that shows the role of syndecan-1 in the pathogenesis of IBD in addition to its relation to CyA and CypA.

## 4. Discussion

Data from this study highlighted the role of syndecan-1 in the pathogenesis and management of IBD in combination with the CypA, CyA complex and probiotics. In comparison to the wild type, the inflammation was more severe in the KO mice, and treatment with CyA led to a greater reduction in inflammation and a greater suppression of expression of immune cell markers. However, the CypA-CyA complex was less effective in controlling the disease and caused a greater exacerbation of inflammation in IL-6, CD147 and CD3 in the KO than in the wild type. On the other hand, when probiotics were added to the CypA-CyA complex, they had less effect in controlling IBD inflammatory markers in the KO compared to more effect in the wild type. Probiotics used alone significantly ameliorated the health status in both strains but more so in the wild type.

In the absence of syndecan-1, the mice were more sensitive to DSS inflammatory stimulation. Actually, the loss of syndecan-1 contributes to epithelial permeability and defect in the barrier, leading to microbiota access to lamina propria and causing a larger chronic and persistent inflammatory reaction and accumulation of inflammatory cells [29]. In its modulation of different proteolytic activities, syndecan-1 plays a crucial role in a plethora of biological processes including cell proliferation, differentiation and redifferentiation [30]. These functions are achieved through its role in integrin activity and migration as well as enhancing the motility of macrophages. Syndecan-1 is also associated with an anti-inflammatory M2 macrophage polarization involved in maintaining the function of the mucosa barrier and the restoration of tight junctions to maintain epithelial cell integrity [29]. The presence of syndecan-1 could also inhibit the secretion of pro-inflammatory cytokines due to its suppressed ectodomain shedding, thus causing an amelioration of intestinal inflammation and neutrophil transmigration [31]. In this study, CyA was very effective in controlling the symptoms, improving and limiting histological alterations, and decreasing pro-inflammatory cytokines expressions. Such effects were more significant in the KO mice. Immunoregulators, including the classical immunosuppressive drug CyA, have been used in IBD therapy, in particular in ulcerative colitis [32,33,34,35]. CyA downregulates the activation of T-lymphocytes by blocking the production of IL-2, inhibits the production of pro-inflammatory cytokines and stimulates apoptosis [32,33,34,35]. Its mechanism of action starts when it binds to CypA, an intracellular binding protein for CyA, forming a Cyp-CypA complex, thus affording anti-inflammatory activity (more so in KO mice) [36]. 

Moreover, the CypA treatment in the syndecan-1 KO mice also showed a decrease in the inflammation originally caused by the DSS, which is contrary to what is being reported in the literature [37,38,39,40,41]. Furthermore, CypA, which is the primary cytosolic binding protein of CyA, was provided intraperitoneally, thus creating a high concentration of extracellular CypA, which can potentially bind to and block CD147 instead of stimulating their chemotactic activity. 

Previous work has proven that pAkt is implicated in IBD pathogenesis where a significant increase in its expression has been reported in a DSS model of colitis [42]. The pAkt, by going back to its normal control levels, deactivated by CypA, showed that CypA could interplay with the Akt pathway and somehow downregulates its activity in both strains. Such an effect is expressed in both strains and slightly more in the KO mice with respect to better tissue repair, more IL-6 suppression, more CD147 suppression and less beta 1 integrins expression, which favors better tissue repair.

Concerning the use of the combination of CyA + CypA, the data of this study showed no added effect in reducing further the inflammatory reaction in the intestines. On the contrary, the inflammation continued in the KO mice with little effect versus a more significant added effect in the wild type as expressed in the lowest pAkt, lowest beta 1 integrins levels, lower CD147 levels and lower IL-6 levels. 

These findings are not all in line with reports from the literature. The maintenance of inflammation in this combination group could probably be ascribed to the excess CypA administered along with the cyclosporine, thus forming a CypA + CyA complex that could probably, in the absence of syndecan-1, stimulate a significant increase in CD3, CD147 and even IL-6. Such changes in the KO group probably activated other signaling pathways, leading to a continuous exacerbation of the disease.

The addition of probiotics to the combination lead to an overall improvement—more so in the wild type than in the syndecan-1 KO mice. Despite the persistent inflammation in the combination with probiotics, the clinical signs and symptoms were improved, which reflects a better preserved intestinal barrier by the presence of probiotics. More investigations are needed to unveil the mechanism of action of the complex in the presence of probiotics.

On the other hand, the use of probiotics in the absence of the CypA-CyA complex revealed a marked reduction in inflammation in both strains but significantly more in the wild-type mice. At the same time, the histology of the sigmoid colon was almost normal and preserved the architecture in 75% of the section, yet very active lymphocyte aggregates existed with a relatively normal epithelial barrier. Regarding the molecular parameters, probiotics alone were more efficient than both drugs in reducing the inflammatory molecules in the colon. This result is in line with previous studies which have evidently shown that probiotics stimulate the differentiation of T-helper 1 cells, boost antibody production, promote the activity of both natural killer cells and phagocytic cells, and increase T-cell apoptosis by inhibiting the transcription of NF-ΚB. In addition, probiotics increase the production of anti-inflammatory cytokines and decrease pro-inflammatory cytokines [43,44,45,46]. Moreover, probiotics prevent the apoptosis of intestinal epithelial cells and stimulate the production of proteins essential for tight junction preservation, thus decreasing the paracellular permeability and restoring barrier function; and the effects of probiotics are in agreement with our results [47,48,49,50]. On the other hand, probiotics produce bacteriocins, thus creating an acidic medium detrimental to pathogenic bacteria yet favorable to the growth of beneficial microorganisms such as lactobacilli and bifidobacteria [51,52,53,54]. Furthermore, it has been shown that probiotics reduce the total T-cells and increase the number of Treg cells in the colonic tissue and blood in addition to enhancing the function of tight junctions [55].

## 5. Conclusions

The potent role of cyclosporine in IBD therapy has been confirmed in both strains as demonstrated by the marked reduction in inflammation taking place particularly in the DSS + CyA group. Altogether, our findings suggest a therapeutic role for CypA in DSS-induced sdc-1 deficient mice. The presence of distinct receptors for extracellular CypA (other than CD147 and sdc-1) on its target cell merits further exploration. However, the low inhibitory effect of inflammation in the group treated with the complex group (CypA-CyA) needs further investigation, since it is recurring in both strains but shows relatively more inhibition in the wild type. Moreover, the effectiveness of probiotics has been clearly revealed when used alone in DSS-induced sdc-1 deficient mice as well as the Balb/c wild type. In contrast, this effectiveness has been partially inhibited in the presence of the CypA-CyA complex. The differential effects of CyA, CypA, probiotics and their combinations on the various inflammatory markers as well as the histological alterations and clinical signs and symptoms speak in favor of an anti-inflammatory role of syndecan-1. However, probiotics need to be considered after more exploration on the mechanisms involved in the presence of CypA and CyA, especially since pAkt was less active in their presence in both strains. Further and deeper investigations need to be carried out to answer the question of how the CypA-CyA complex decreases the anti-inflammatory effect of probiotics and ascertain the likely mechanisms involved.

Lastly, this study has a limitation with respect to the number of animals. Actually, the syndecan-1 KO mice were difficult to breed and achieved good numbers of progenies. This led to a decrease in the types of groups, whereby a group of CyA with probiotics and a group of CypA with probiotics could have been added in order to have a more complete picture. Another limitation included the study of other inflammatory markers like TGFβ and TNFα, which could have been useful in exploring other relevant signaling pathways that are probably involved in the pathogenic process.

## Figures and Tables

**Table 1:** Various groups and corresponding treatments.

Balb/C Mice—Control Group Given Normal Water	Syndecan-1 Null Mice—Control GROUP Given Normal Water
Balb/C mice + DSS only	Syndecan-1 null mice + DSS only
Balb/C mice + DSS + CypA	Syndecan-1 null mice + DSS + CypA
Balb/C mice + DSS + CyA	Syndecan-1 null mice + DSS + CYA
Balb/C mice + DSS + CypA + CyA	Syndecan-1 null mice + DSS + CypA + CyA
Balb/C mice + DSS + CypA + CyA + Probiotics	Syndecan-1 null mice + DSS + CypA + CyA + Probiotics
Balb/C mice + DSS + Probiotics	Syndecan-1 null mice + DSS + Probiotics

CyA: cyclosporine A; CypA: cyclophilin A; DSS: dextran sodium sulfate.

**Table 2:** Scoring system used to evaluate the histological alterations in dextran sulfate sodium (DSS)-induced colitis [27].

Feature	Score	Description
Severity of inflammation	0	None
	1	Mild
	2	Moderate
	3	Severe
Extent of inflammation	0	None
	1	Mucosa
	2	Mucosa and submucosa
	3	Transmural
Crypt damage	0	None
	1	1/3 damaged
	2	2/3 damaged
	3	Crypts lost, surface epithelium present
	4	Crypt and surface epithelium lost

**Table 3:** Comparative assessment of clinical symptoms and signs.

	Wild-Type Balb/C		Syndecan-1 KO	
Groups	Diarrhea	Hematochezia	Diarrhea	Hematochezia
1-Control	0	0	0	0
2-DSS only	6	5	8	6
3-DSS + CypA	5	5	7	5
4-DSS + CyA	4	3	3	4
5-DSS + CypA + CyA	6	3	3	4
6-DSS + CypA + CyA + probiotics	4	2	3	3
7-DSS + probiotics	2	0	2	0

Each group consists of 8 animals.

**Table 4:** Comparative assessment of histological alterations in the colon of both strains of experimental mice as a result of H&E and PAS stains.

Group Number	Syndecan-1 Null Mice	Balb/C Mice
1—Control	Normal architecture, normal crypts, continuous epithelial lining, normal distribution of goblet cells and elongated crypts—no inflammatory infiltrate	Normal architecture, normal crypts, continuous epithelial lining, normal distribution of goblet cells and elongated crypts—no inflammatory infiltrate
2—DSS only	Epithelial loss, loss of crypts, cryptitis, massive infiltration of inflammatory cells and invasion of the mucosa and submucosa and more edema between mucosa and submucosa, loss of 80–90% of goblet cells	Epithelial loss, loss of crypts, cryptitis, infiltration of inflammatory cells, almost complete depletion of goblet cells (100%)
3—DSS + CypA	Mild inflammation, 1/3 damaged crypts—low edema between mucosa and submucosa, presence of 75% of goblet cells partial resorption of mucosa and submucosa	High inflammatory infiltrate—disorganized crypts and partial loss of epithelial lining and loss of more than 60% of goblet cells
4—DSS + CyA	Limited infiltration of inflammatory cells—partial restoration of the epithelial lining, mucosal architecture and crypts integrity, around 75% of goblet cells are still present	Limited infiltration of inflammatory cells-Partial effect on epithelial lining—restoration of mucosal architecture and crypts integrity, >70% of goblet cells present
5—DSS + CypA + CyA	2/3 of the crypts are lost, intensive infiltration in mucosa and submucosa, epithelia loss in most of the places, depletion of goblet cells	Moderate restoration of the majority of mucosal architecture and crypts, loss of 50% of goblet cells
6—DSS + CypA + CyA + probiotics	Less effective than in wild-type mice—mild progress, crypts improved, partial relief-moderate inflammation and loss of 75% of goblet cells	Mild inflammation–restoration of the majority of mucosal architecture and crypts with loss of 50% of goblet cells
7-DSS + probiotics	Some improvement, clear restoration of mucosal architecture, moderate infiltration, less improvement than in wild type, same effect on goblet cells, 75% of cells were restored	Limited infiltration: mild to no inflammation in some areas, restoration of the majority of the mucosal architecture and epithelial lining—very effective–high improvement, of goblet cells with 80% restored

**Table 5:** Comparative effect of cyclosporine or cyclophilin on the various parameters in both strains.

Outcomes	Syndican-1/KO	Wild Type
Clinical signs and symptoms	More severe	Less severe
Histological alterations	More severe	Less severe
IL-6	More inhibition	Less inhibition
C147	More inhibition	Less inhibition
CD3	More inhibition	Less inhibition
β1 integrin	Less inhibition	More inhibition
pAKT	Less inhibition	More inhibition

**Table 6:** Comparative effect of Probiotics during the CYA-CYPA combination.

Outcomes	Syndican-1/KO	Wild Type
Clinical signs and symptoms	Slightly more severe	Same or less
Histological alterations	More severe	Less severe
IL-6	Less effect/More IL-6 expression	More effect/Less IL-6 expression
C147	Less effect/More C147 expression	More effect/Less C147 expression
CD3	Less effect/More CD3 expression	More effect/Less CD3 expression
β1 integrin	Less effect/More β1 Integrin expression	More effect/Less β1 Integrin expression
pAKT	Less effect/More pAKT activity	More effect/Less pAKT activity

## Data Availability

Data contained within the article.
